# Prometheus’s Fire, Part I: The Flight of Icarus—Molecular Mechanisms Underlying Adipose Tissue Dysfunction in Obesity: From Adaptive Hyperplasia to Pathological Hypertrophy

**DOI:** 10.3390/ijms27209055

**Published:** 2026-10-13

**Authors:** Anna Grudniewska, Beata Mrozikiewicz-Rakowska, Kacper Wiszniewski, Wojciech Bik, Ilona Szabłowska-Gadomska, Alina Kuryłowicz

**Affiliations:** 1 Lux Med Medical Centre in Warsaw, 49 Komitetu Obrony Robotników Street, 02-146 Warsaw, Poland; grudniewska.anna@gmail.com; 2 Department of Endocrinology, Centre of Postgraduate Medical Education, Marymoncka St. 99/103, 01-813 Warsaw, Poland; kacpor94@gmail.com; 3 Department of Neuroendocrinology, Centre of Postgraduate Medical Education, Marymoncka St. 99/103, 01-813 Warsaw, Poland; wojciech.bik@cmkp.edu.pl; 4 Laboratory for Cell Research and Application, Medical University of Warsaw, 02-106 Warsaw, Poland; ilona.szablowska-gadomska@wum.edu.pl; 5 Department of General Medicine and Geriatric Cardiology, Medical Centre of Postgraduate Education, 00-401 Warsaw, Poland; alina.kurylowicz@cmkp.edu.pl

**Keywords:** obesity, adipose tissue dysfunction, adipose tissue expandability, adipocyte hypertrophy, adipocyte hyperplasia, metaflammation, NLRP3 inflammasome, adipokines, immunometabolism, insulin resistance

## Abstract

Obesity is a heterogeneous disease in which cardiometabolic risk is shaped not only by adipose tissue mass but also by the capacity of adipose tissue to expand and remain metabolically functional. This narrative review provides an integrated, evidence-weighted molecular framework, the central original contribution of this work, for understanding how initially adaptive adipose tissue expansion transitions into pathological hypertrophy and self-sustaining immunometabolic dysfunction. We examine how impaired adipogenesis, angiogenesis and extracellular-matrix remodelling converge with mitochondrial and endoplasmic reticulum stress, oxidative stress, hypoxia and lipotoxicity to destabilise adipocyte homeostasis. Particular attention is given to the NLRP3 inflammasome, positioned within a broader network including JNK, IKKβ/NF-κB, TLR4 and cGAS–STING signalling, as a molecular integrator of metabolic danger signals. We further explore how adipokine remodelling, loss of adiponectin-mediated protection and enhanced leptin signalling interact with immune-cell recruitment to establish self-amplifying inflammatory circuits. Evidence is stratified across experimental, translational and human levels; a summary table maps each mechanism to its strength of evidence and current clinical relevance. This framework explains metabolic phenotype heterogeneity among individuals with comparable adiposity and establishes the molecular foundation for Parts II and III of the Prometheus’s Fire series.

## 1. Introduction

Obesity is a major global health challenge of the twenty-first century, affecting more than one billion people worldwide [1] and substantially increasing the risk of type 2 diabetes mellitus, cardiovascular disease, metabolic dysfunction-associated steatotic liver disease, several malignancies and premature mortality. Although obesity has traditionally been viewed primarily as a consequence of excessive adipose tissue accumulation, accumulating evidence suggests that metabolic risk is influenced not only by the amount of adipose tissue but also by its capacity to expand, remodel and maintain metabolic homeostasis. This has encouraged a broader view of obesity in which adipose tissue function is considered alongside adipose tissue quantity.

Adipose tissue is now recognised as a dynamic endocrine and immunometabolic organ that contributes to whole-body energy homeostasis through continuous interactions among adipocytes, immune cells, stromal and vascular components, and the extracellular matrix. Under physiological conditions, excess energy can be accommodated through adaptive tissue expansion, including the recruitment and differentiation of new adipocytes. This process helps preserve cellular integrity, insulin sensitivity and endocrine function. However, when this adaptive capacity becomes increasingly constrained, further tissue expansion may depend predominantly on adipocyte hypertrophy and may become accompanied by cellular stress, impaired tissue remodelling, low-grade inflammation and progressive metabolic dysfunction [2].

Growing evidence indicates that adipocyte hypertrophy is not simply a morphological consequence of obesity but contributes to the molecular processes linking impaired adipose tissue plasticity with systemic metabolic dysfunction. Hypertrophic adipocytes are associated with mitochondrial dysfunction, endoplasmic reticulum stress, oxidative stress, local hypoxia and altered adipokine secretion. These disturbances can activate interconnected stress-responsive pathways, including c-Jun N-terminal kinase (JNK), inhibitor of nuclear factor κB kinase β (IKKβ), nuclear factor κB (NF-κB) and the NLRP3 inflammasome. Rather than operating independently, these mechanisms may reinforce one another, promoting inflammatory signalling, immune-cell recruitment, impaired insulin action and further deterioration of adipose tissue function [3].

Despite substantial advances in obesity research, the individual components of adipose tissue dysfunction, including impaired adipogenesis, hypoxia, organelle stress, adipokine dysregulation and innate-immune activation, are already well established. The principal contribution of this review is therefore not the proposal of a new isolated pathway, but the integration of these processes into an evidence-weighted model of progressive loss of adipose tissue adaptive capacity. Within this model, the ‘expandability threshold’ is used as a conceptual, depot- and individual-dependent boundary rather than a fixed biological cut-off, and NLRP3 is examined as one important inflammatory node within a broader stress-response network.

The present state-of-the-art narrative review organises current molecular, experimental and translational evidence around this integrative framework: limited adipogenic, vascular and extracellular-matrix adaptation increases reliance on adipocyte hypertrophy; hypertrophy is associated with lipotoxic, hypoxic, mitochondrial and endoplasmic reticulum stress; and these disturbances engage overlapping stress-responsive and innate-immune pathways that promote immune-cell recruitment, adipokine remodelling and insulin resistance. These processes can coexist rather than occur as a strictly linear sequence, and their relative contribution varies by adipose depot, sex, age, genetic background and stage of obesity. Throughout this review, ‘adipose tissue quality’ refers to the composite biological state of the tissue, including adipocyte size and expandability, adipogenic capacity, vascularisation, extracellular-matrix remodelling and fibrosis, inflammatory activity, adipokine profile, lipid-buffering capacity and insulin-responsive metabolic function. This biological construct is distinguished from the more limited set of parameters currently measurable in clinical practice or research.

As the first article in the Prometheus’s Fire review series, the present paper establishes the molecular framework for the subsequent parts of the series. Part II, ‘The Hydra of Inflammation’, extends this perspective to the reciprocal inflammatory interactions between dysfunctional adipose tissue and peripheral organs, whereas Part III, ‘The Forge of Hephaestus’, examines pharmacological and non-pharmacological strategies capable of modifying these interconnected processes. Together, the three articles follow a continuum from the molecular origins of adipose tissue dysfunction, through its propagation across organs, to the therapeutic opportunities for restoring immunometabolic homeostasis.

**Literature Search and Review Approach.** PubMed/MEDLINE and Scopus were searched for publications from January 2019 through August 2026. Searches combined terms related to adipose tissue expandability and hypertrophy, adipogenesis, adipose tissue dysfunction and inflammation, NLRP3 inflammasome, metaflammation, mitochondrial and endoplasmic reticulum stress, adipokines, adiponectin, leptin, metabolically healthy obesity, cGAS–STING, pyroptosis, lipotoxicity, adipose tissue hypoxia and crown-like structures. No language restriction was applied. Eligible evidence comprised original mechanistic studies, human observational and translational studies, randomised clinical trials, and systematic or high-quality narrative reviews directly addressing the cellular, molecular or immunometabolic mechanisms of adipose tissue dysfunction. Reports were deprioritised or excluded when they lacked direct relevance to adipose tissue biology, provided insufficient methodological detail, duplicated evidence already represented by a stronger or more recent source, or addressed obesity-related inflammation without informing the proposed mechanistic framework. Seminal pre-2019 studies were retained when they established foundational mechanisms, including adipose tissue macrophage infiltration [4,5], NLRP3 inflammasome activation in obesity [6,7], adipose tissue endocrine function [8] and transcriptional regulation of adipogenesis [9,10]. Evidence was synthesised thematically rather than using a formal systematic-review protocol. When findings were inconsistent, greater interpretive weight was given to human data, replicated findings, studies with stronger design and phenotyping, and evidence supported across more than one experimental level; discordant findings and important model-dependent limitations are identified in the relevant sections. PRISMA methodology was not applied because this work is a narrative, state-of-the-art review rather than a systematic review or meta-analysis.

## 2. Does Excessive Adipose Tissue Accumulation Invariably Lead to Subclinical Inflammation?

Excess adipose tissue accumulation does not produce a uniform inflammatory or metabolic phenotype. Individuals with similar adiposity may differ substantially in fat distribution, adipocyte morphology, adipogenic capacity and inflammatory activity, supporting the view that tissue function and distribution provide information beyond total fat mass alone [11,12,13,14].

Metabolically healthy and metabolically unhealthy obesity illustrate this heterogeneity, although these phenotypes are dynamic rather than fixed. Their differences cannot be attributed to a single mechanism and likely reflect interacting effects of adipose depot, ectopic lipid deposition, age, sex, genetics, lifestyle and tissue remodelling [11,12,13,14,15].

Adipose tissue is therefore considered here as a dynamic endocrine, metabolic and immune organ in which adipocytes, immune cells, vascular structures and extracellular matrix components jointly regulate lipid storage, endocrine signalling, insulin sensitivity and local inflammatory tone [4,5,12,14,16,17].

Hyperplasia and hypertrophy should not be interpreted as mutually exclusive or obligatorily sequential states. They may coexist within the same individual and differ across subcutaneous and visceral depots. The working model is that metabolic risk tends to increase when adaptive recruitment of new adipocytes, vascularisation and matrix remodelling become insufficient relative to lipid-storage demand, increasing reliance on hypertrophic expansion [6,7,13,16,18,19].

The adaptive-to-dysfunctional transition used in this review is therefore a conceptual framework, not a universal linear trajectory. Its timing and dominant mechanisms vary by depot, individual and stage of obesity.

## 3. Adaptive Adipose Tissue Expansion: From Hyperplasia to Hypertrophy

Adipose tissue can accommodate sustained energy surplus through both recruitment of new adipocytes and enlargement of existing cells. Hyperplastic expansion is generally considered more metabolically adaptive because it distributes lipids across a larger number of adipocytes, whereas disproportionate hypertrophy is associated with impaired metabolic flexibility [13,16,20,21].

Adipogenesis is coordinated principally by PPARγ and C/EBPα and depends on adequate progenitor availability, angiogenesis and extracellular-matrix remodelling [9,10,21,22,23]. Each of these determinants varies between depots and individuals and helps explain why hyperplasia and hypertrophy can coexist to different degrees.

When lipid-storage demand increasingly exceeds adipogenic, vascular or structural adaptation, enlargement of existing adipocytes becomes more prominent. Rather than a single fixed threshold, this represents a gradual and context-dependent shift in tissue remodelling [13,16,18].

Hypertrophy is clinically relevant because larger adipocytes are associated with impaired lipid buffering, altered endocrine function and cellular stress [6,7,18]. These downstream hypoxic, mitochondrial, endoplasmic-reticulum and inflammatory mechanisms are summarised in Section 4 and examined in mechanistic detail in Section 7.

The mode of tissue expansion thus shapes the inflammatory and metabolic consequences of obesity, while the relative balance between hyperplasia and hypertrophy remains depot- and context-specific.

This variable balance provides one biological explanation for metabolic heterogeneity among individuals with similar levels of adiposity, while remaining only one component of a broader multifactorial risk architecture.

For this reason, the term ‘expandability threshold’ is used in this review as a conceptual shorthand for the point at which storage demand exceeds the adaptive capacity of a given depot, not as a clinically measurable or universally discrete cut-off.

## 4. Molecular Transition from Adipocyte Hypertrophy to Immunometabolic Dysfunction

Adipocyte hypertrophy is associated with a network of interdependent stress responses rather than a single dominant inflammatory pathway. Lipotoxicity, hypoxia, mitochondrial and endoplasmic reticulum stress and oxidative stress can engage JNK, IKKβ/NF-κB, TLR4 and inflammasome signalling, while mitochondrial damage may also activate cGAS–STING pathways. These systems interact with adipokine remodelling and immune-cell recruitment to impair insulin signalling and tissue homeostasis [6,7,8,14,18,19,24,25,26,27,28].

Persistent lipid overload increases FFAs, ceramides and ROS, compromises mitochondrial and ER homeostasis and, when vascular adaptation is inadequate, promotes local hypoxia and HIF-1α-dependent responses [6,7,8,16,18,19,24]. These stressors are best viewed as partially overlapping inputs into a wider inflammatory network; their relative importance differs by model, tissue depot and disease stage.

The human evidence base is strongest for the phenotypic links among adipocyte hypertrophy, visceral adiposity, altered adipokine profiles, macrophage accumulation, systemic inflammation and insulin resistance [4,5,11,12,13,14,29,30,31]. Mitochondrial dysfunction, endoplasmic reticulum stress, hypoxia and NLRP3-associated signalling are supported by convergent human tissue observations and experimental studies, but causal ordering and quantitative contribution in humans remain incompletely defined [6,7,18,25,26]. By contrast, detailed mechanisms involving TXNIP-dependent inflammasome assembly, gasdermin-D-mediated pyroptosis in adipocytes and cGAS–STING signalling in obesity are derived predominantly from cellular and animal models, with human translational validation still limited [27,28,32,33,34]. The mechanistic sequence below should therefore be read as an evidence-weighted framework rather than as a uniformly proven causal cascade in humans.

JNK, IKKβ/NF-κB and TLR4 signalling provide major links between metabolic stress, inflammatory gene expression and impaired insulin action [6,7,14,18]. NLRP3–caspase-1 signalling represents an additional integrative node that can process IL-1β and IL-18 and amplify inflammatory responses, but it operates alongside rather than above these pathways [6,7,25,26]. cGAS–STING is included as an emerging mitochondrial danger-sensing pathway for which obesity-specific human evidence remains less mature [27,28].

The detailed molecular architecture of NLRP3 activation, DAMP sensing, caspase-1 and pyroptosis is therefore reserved for Section 7. No single pathway accounts for the full inflammatory phenotype of dysfunctional adipose tissue.

At tissue level, altered adipokine secretion and recruitment of monocytes/macrophages further reinforce this network. Reduced adiponectin, increased leptin and pro-inflammatory mediators, and crown-like structures around damaged adipocytes are associated with dysfunctional adipose tissue, while macrophage states in vivo form a heterogeneous continuum rather than a strict M1/M2 binary [4,5,12,14,35].

**Figure 1:**
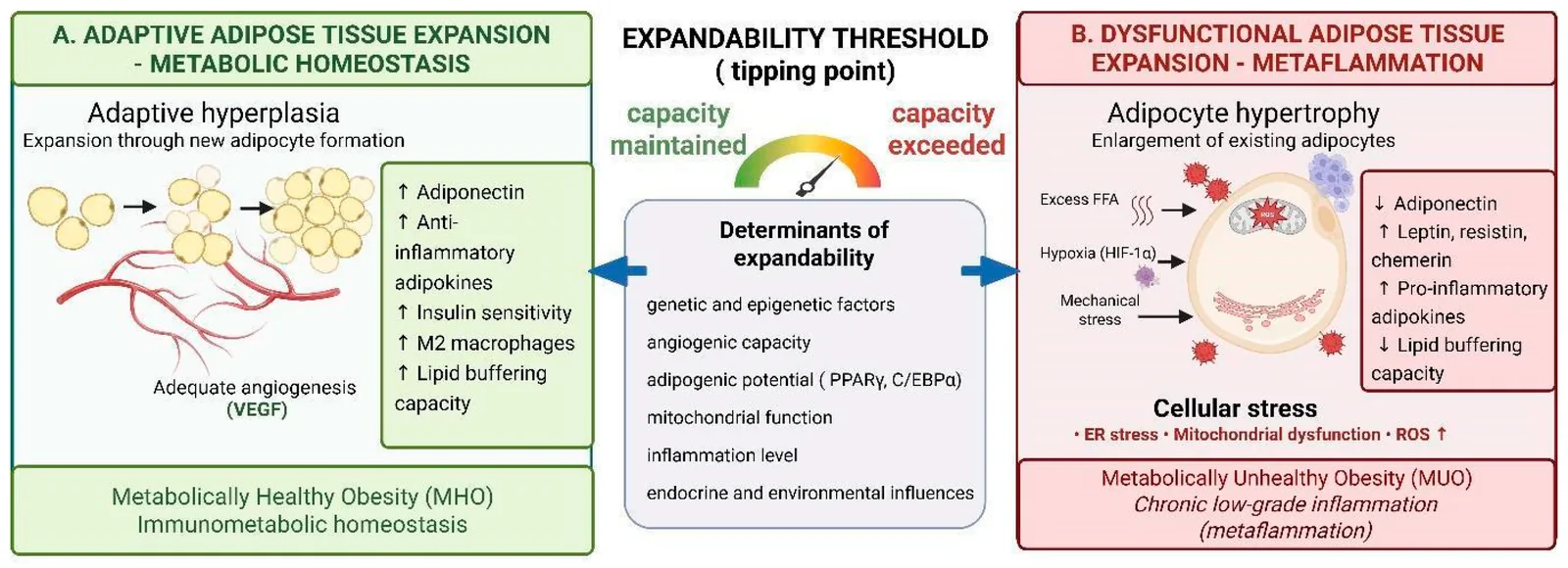

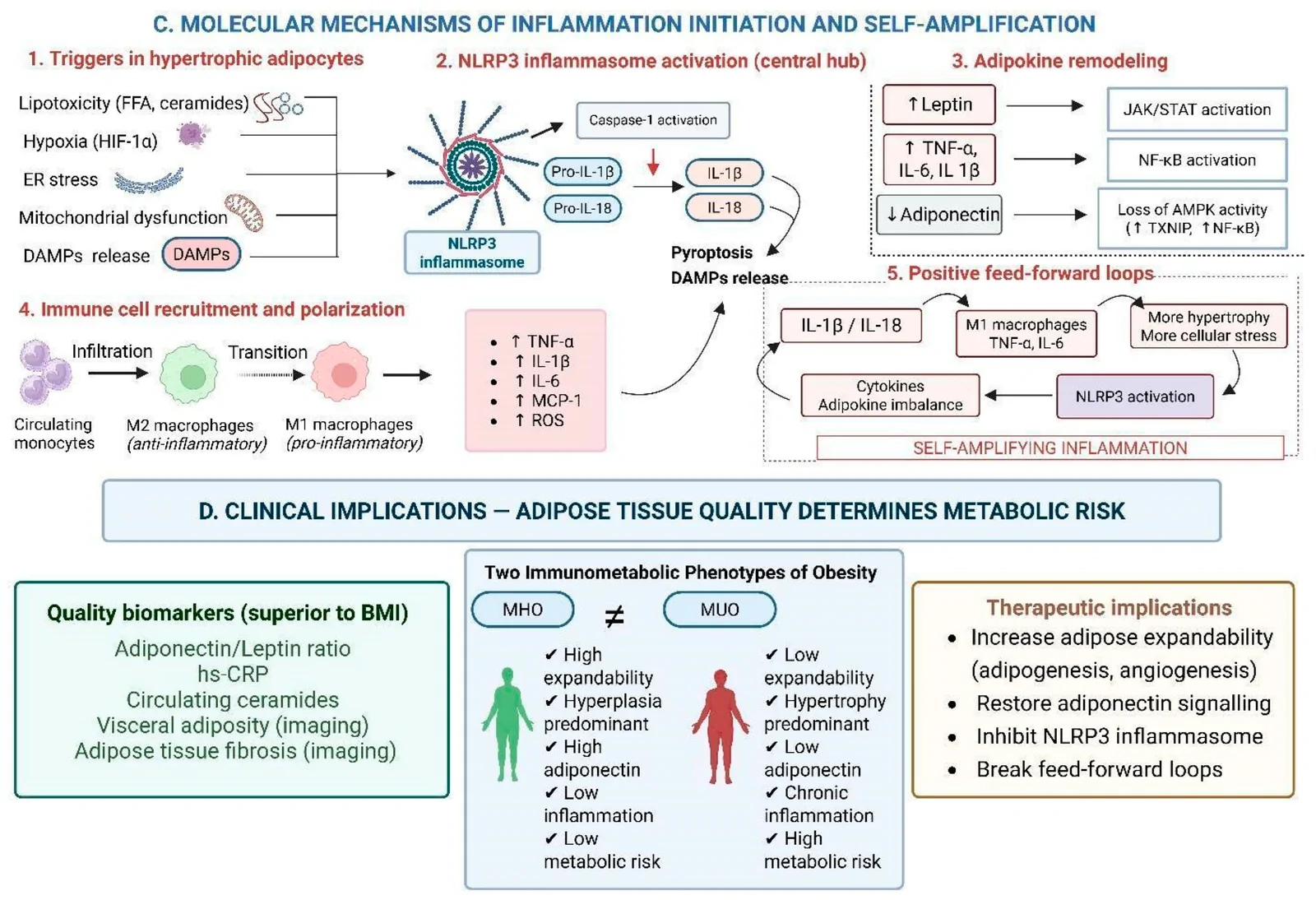
**Adipose tissue expandability as a determinant of immunometabolic dysfunction in obesity.** Adaptive adipose tissue expansion through hyperplasia preserves tissue homeostasis through coordinated adipogenesis, adequate vascularisation, a predominantly anti-inflammatory immune environment and maintained adiponectin secretion. In contrast, impaired expandability promotes adipocyte hypertrophy, cellular stress, hypoxia, lipotoxicity and adipokine dysregulation, leading to metaflammation and progressive immunometabolic dysfunction. NLRP3 inflammasome activation and related feed-forward inflammatory mechanisms may further reinforce this process. The figure also highlights selected biomarkers of adipose tissue quality and potential therapeutic targets. Created in BioRender. Grudniewska, A. (2026) https://BioRender.com/6h1nbyf (accessed on 6 August 2006). **Abbreviations:** AMPK, AMP-activated protein kinase; BMI, body mass index; C/EBPα, CCAAT/enhancer-binding protein alpha; DAMP, damage-associated molecular pattern; ER, endoplasmic reticulum; FFA, free fatty acid; HIF-1α, hypoxia-inducible factor 1 alpha; hs CRP, high-sensitivity C-reactive protein; IKKβ, inhibitor of nuclear factor kappa B kinase beta; IL-1β, interleukin-1 beta; IL 6, interleukin-6; IL-18, interleukin-18; JAK2, Janus kinase 2; M1/M2, macrophage polarisation phenotypes; MHO, metabolically healthy obesity; MUO, metabolically unhealthy obesity; NF-κB, nuclear factor kappa B; NLRP3, NOD-like receptor family pyrin domain containing 3; OB-Rb, long isoform of the leptin receptor; PPARγ, peroxisome proliferator-activated receptor gamma; ROS, reactive oxygen species; STAT3, signal transducer and activator of transcription 3; TNF α, tumour necrosis factor alpha; TXNIP, thioredoxin-interacting protein; VEGF, vascular endothelial growth factor.

Fibrotic extracellular-matrix remodelling reduces tissue compliance and adipogenic plasticity, linking structural remodelling with impaired lipid buffering [8,16,24]. Figure 1 summarises these interacting domains without implying a universal temporal order.

## 5. Determinants of Adipose Tissue Expandability: Why Does Adipose Tissue Become Dysfunctional?

The capacity of adipose tissue to accommodate excess energy varies across individuals and depots, reflecting a convergence of developmental, genetic, vascular, endocrine and environmental determinants. The following subsections examine each of these factors in turn.

### 5.1. Developmental and Genetic Determinants

Adipogenic capacity varies substantially among individuals. During childhood and adolescence, adipose tissue retains a relatively high progenitor capacity, which declines with advancing age and accumulating metabolic stress. Genetic variation in PPARγ activity, adipokine signalling and progenitor responsiveness contributes to interindividual differences in expandability [9,10,13,36,37].

Interindividual variation in adipogenic capacity, adipocyte differentiation, extracellular-matrix remodelling and vascular adaptation contribute to interindividual differences in adipose tissue expandability [9,13,20,21,22,23,38]. These mechanisms probably contribute to the well-recognised phenomenon of metabolically healthy obesity (MHO), although the molecular basis underlying this phenotype remains incompletely understood [4,13,14].

### 5.2. Angiogenesis as a Limiting Factor for Adipose Tissue Expansion

Among the factors governing adipose tissue expandability, vascular adaptation appears particularly critical. Hyperplastic expansion requires coordinated angiogenesis capable of supplying newly formed adipocytes with oxygen and nutrients. Vascular endothelial growth factor (VEGF)-dependent vascular remodelling therefore represents an essential prerequisite for maintaining tissue homeostasis during chronic energy excess [16,22,23].

When angiogenesis fails to keep pace with adipose tissue growth, oxygen diffusion becomes progressively impaired, leading to HIF-1α activation, extracellular-matrix remodelling and fibrosis [8,24,39]. Fibrosis not only reflects tissue damage but also further restricts adipogenesis, creating a self-reinforcing cycle in which impaired vascular adaptation accelerates hypertrophic expansion and chronic inflammation.

### 5.3. Endocrine and Metabolic Regulation of Adipose Tissue Plasticity

Hormonal signals continuously modulate adipose tissue expandability. Under physiological conditions, insulin, adiponectin and PPARγ-dependent signalling promote adipocyte differentiation and preserve metabolic flexibility [10,13,16,40]. Conversely, insulin resistance, chronic hyperinsulinaemia and inflammatory cytokines progressively impair adipogenesis while simultaneously increasing lipid influx into existing adipocytes.

This endocrine shift transforms adipose tissue from an efficient lipid-buffering organ into a metabolically overloaded tissue characterised by enhanced lipolysis, altered adipokine secretion and increasing inflammatory activity [12,13,14,41]. Consequently, endocrine dysregulation should be considered both a consequence and an important driver of adipose tissue dysfunction.

### 5.4. Lifestyle and Environmental Factors

Environmental influences substantially modify the biological expandability of adipose tissue. Persistent caloric excess, diets rich in saturated fatty acids, physical inactivity and chronic metabolic stress accelerate exhaustion of adipogenic capacity and favour hypertrophic remodelling [13,16,18,42]. Conversely, regular physical activity can improve adipose tissue vascularisation and angiogenic capacity and may support adipose tissue plasticity [42,43].

Chronic inflammation itself becomes an active determinant of tissue remodelling. Pro-inflammatory cytokines released by hypertrophic adipocytes and infiltrating macrophages suppress adipocyte differentiation, thereby further limiting hyperplastic expansion [6,7,14,18]. This establishes a vicious cycle in which inflammation inhibits adipogenesis, impaired adipogenesis promotes hypertrophy, and hypertrophy amplifies inflammation.

### 5.5. Adipose Tissue Expandability as an Integrative Concept

Adipose tissue expandability thus reflects the combined output of developmental, genetic, vascular, endocrine and environmental determinants, with depot- and individual-specific limits that cannot currently be measured by a single validated test.

**Figure 2:**
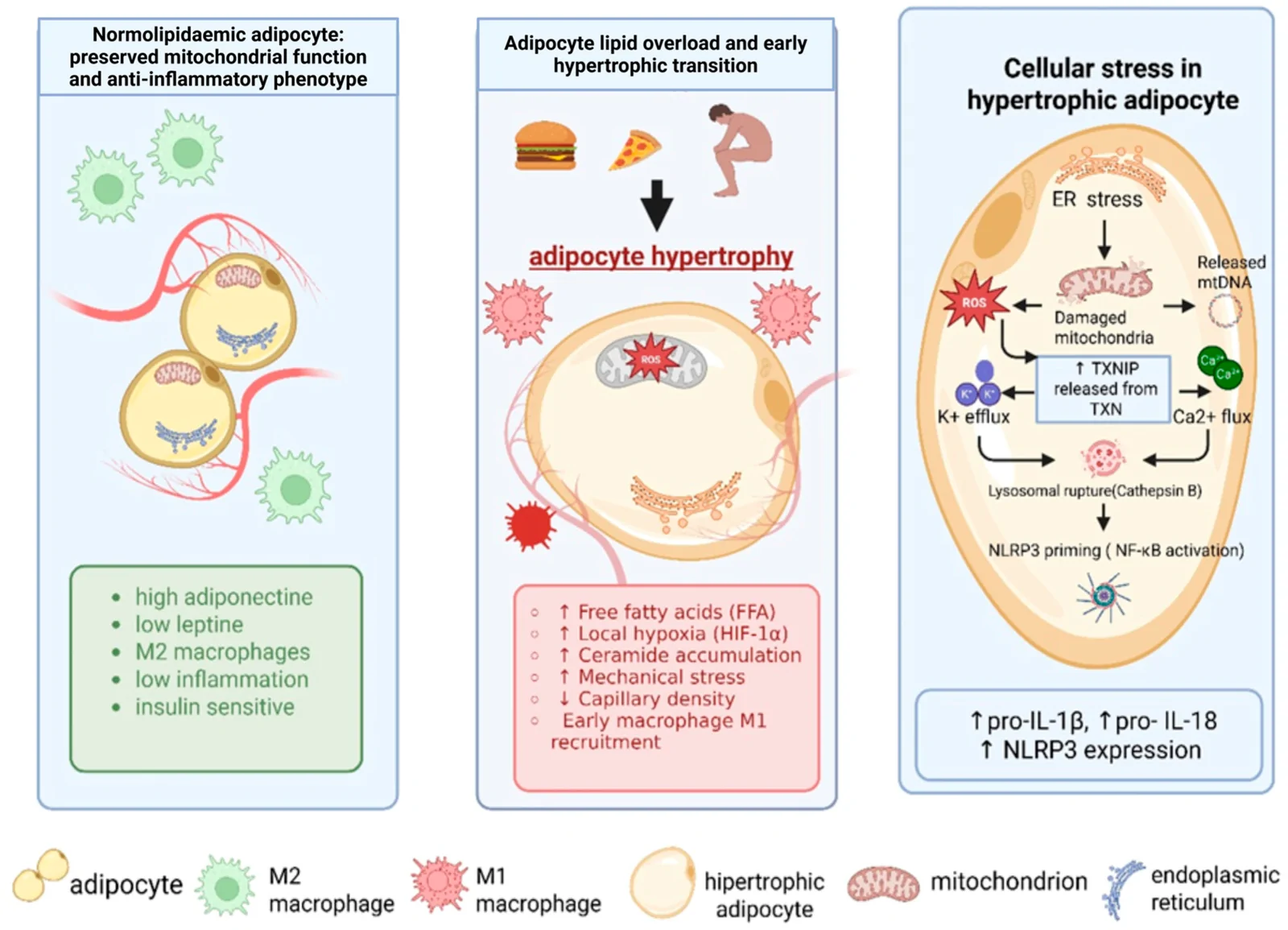
**Adipocyte hypertrophy and cellular stress in the initiation of NLRP3 inflammasome activation in obese adipose tissue.** Adipocyte hypertrophy is associated with hypoxia, lipotoxicity and endoplasmic reticulum stress, which promote mitochondrial dysfunction, reactive oxygen species generation and inflammatory signalling. These disturbances contribute to NLRP3 inflammasome priming and activation through interconnected mechanisms involving NF-κB activation, TXNIP, ionic imbalance and mitochondrial damage. The resulting inflammatory response may further promote adipose tissue dysfunction and contribute to obesity-associated metaflammation. Created in BioRender. Grudniewska, A. (2026) https://BioRender.com/lvxqv78 (accessed on 15 July 2026). **Abbreviations:** ATP, adenosine triphosphate; Ca^2+^, calcium ion; ER, endoplasmic reticulum; FFA, free fatty acid; HIF-1α, hypoxia-inducible factor 1 alpha; IP3R, inositol 1,4,5-trisphosphate receptor; K^+^, potassium ion; LRR, leucine-rich repeat; M1/M2, macrophage polarisation phenotypes; mtDNA, mitochondrial DNA; NF-κB, nuclear factor kappa B; NLRP3, NOD, LRR and pyrin domain-containing protein 3; P2X7, purinergic receptor P2X7; ROS, reactive oxygen species; TLR4, Toll-like receptor 4; TXNIP, thioredoxin-interacting protein.

Figure 2 illustrates representative cellular signals that can accompany hypertrophic remodelling, including hypoxia, lipotoxicity, organelle stress, ROS generation and innate-immune activation. The figure highlights NLRP3 as one convergence mechanism, while the detailed inflammasome sequence is discussed in Section 7 to avoid duplication.

These determinants support the use of adipose tissue expandability as an integrative biological concept rather than a directly measurable clinical trait. Its relative contribution is likely to differ across depots and individuals, and the molecular boundary between adaptive remodelling and persistent dysfunction remains insufficiently defined in humans.

## 6. Clinical Implications: From Adipose Tissue Mass to Adipose Tissue Function

For clinical translation, the biological concept of ‘adipose tissue quality’ should be separated from what can currently be measured. In this review, tissue quality encompasses adipocyte size and expandability, adipogenesis, vascularisation, fibrosis and extracellular-matrix remodelling, inflammatory activity, adipokine profile, lipid-buffering capacity and insulin-responsive metabolic function. No single clinical test captures this multidimensional construct. BMI, waist circumference and standard metabolic risk factors remain established and accessible parameters, while selected imaging and circulating biomarkers assess only individual components of adipose tissue phenotype [11,13].

The proposed markers are also at different stages of validation. Visceral adiposity assessed by CT or MRI has substantial human outcome evidence and can refine phenotyping when imaging is otherwise indicated, but is not a direct measure of tissue function. Perivascular adipose-tissue imaging and imaging of fibrotic/remodelled adipose tissue remain predominantly specialist or research approaches [31]. The adiponectin-to-leptin ratio is a promising research biomarker associated with insulin resistance and adipose dysfunction [29,30], but lacks standardised clinical thresholds and proven incremental utility. Circulating ceramides are the subject of a growing prognostic literature in cardiometabolic disease, but they are not validated as a specific measure of adipose tissue quality and their availability is limited. Inflammasome-related markers, DAMPs and molecular stress signatures remain experimental for obesity risk stratification.

Therapeutically, weight reduction and improvement in established cardiometabolic risk factors have demonstrated clinical benefit. Some approved interventions may also improve adipocyte size, adipokine profiles, insulin sensitivity or ectopic lipid handling, but the extent to which clinical outcomes are mediated specifically by restoration of adipose tissue function is uncertain. Strategies directly targeting adipogenesis, fibrosis, mitochondrial stress, inflammasome activity or other components of the proposed quality framework should therefore be distinguished from established treatment effects when they remain mechanistic or experimental [6,7,13,18,44].

At present, no validated clinical method directly quantifies adipose tissue expandability or identifies a universal boundary between adaptive and pathological remodelling. Translational priorities include standardisation of candidate measures and prospective testing of whether they improve prediction or treatment selection beyond conventional variables.

The clinical value of this framework is currently conceptual and hypothesis-generating: it identifies measurable dimensions of tissue dysfunction that may eventually complement, but should not replace, established obesity and cardiometabolic risk assessment.

## 7. Molecular Mechanisms of the Self-Amplifying Inflammatory State in Adipose Tissue and Target Organs in Obesity

The adipocyte stress responses and adipokine changes described in earlier sections can initiate a self-amplifying immunometabolic state [6,7,18]. A key feature of this system is the mutual amplification of signals: the products of inflammation become new stimuli (DAMPs) for the same signalling pathways, whilst organ dysfunction in peripheral tissues provides additional factors that sustain inflammation within adipose tissue [25,26]. The following section presents aspects of the molecular mechanisms underlying this coupling.

### 7.1. The NLRP3 Inflammasome: A Molecular Hub Linking Metabolic Stress to Chronic Immunometabolic Inflammation

The NLRP3 inflammasome is a cytosolic multiprotein signalling platform that functions as a central component of the innate immune response by sensing intracellular danger signals associated with metabolic stress and cellular damage [6,7,25,26]. Rather than acting as a constitutively active inflammatory complex, the inflammasome is assembled only after sequential integration of multiple stress-related stimuli, thereby providing a tightly regulated mechanism that links metabolic disturbances with inflammatory activation.

The canonical NLRP3 inflammasome consists of three functional components: the sensor protein NLRP3 (NOD-, LRR- and pyrin domain-containing protein 3), the adaptor protein apoptosis-associated speck-like protein containing a caspase recruitment domain (ASC), and the effector protease caspase-1. Each component performs a distinct but complementary role, ensuring that inflammasome activation occurs only under conditions of sufficient cellular stress [6,7,25,26].

NLRP3 serves as the intracellular sensor responsible for detecting a broad spectrum of danger-associated molecular patterns (DAMPs) generated during adipocyte dysfunction. Structurally, the protein comprises three major domains: an N-terminal pyrin domain (PYD), a central nucleotide-binding and oligomerisation (NACHT) domain, and a C-terminal leucine-rich repeat (LRR) domain. The PYD mediates interaction with ASC, the NACHT domain drives ATP-dependent oligomerisation of the receptor during activation, whereas the LRR domain is believed to participate in autoregulation and sensing of metabolic danger signals [6,7,25,26,32]. Current evidence suggests that NLRP3 does not recognise a single ligand directly; instead, it integrates diverse intracellular disturbances that collectively indicate loss of cellular homeostasis.

ASC functions as the essential molecular bridge between the activated NLRP3 receptor and procaspase-1. Owing to the presence of both a pyrin domain (PYD) and a caspase recruitment domain (CARD), ASC links sensor activation with downstream effector signalling. Upon inflammasome activation, ASC molecules undergo rapid oligomerisation into a characteristic supramolecular structure, commonly referred to as the ASC speck, which serves as a highly efficient signalling platform for caspase-1 activation [25,26].

The final component of the complex, procaspase-1, is recruited to the ASC scaffold through CARD–CARD interactions. Subsequent autocatalytic cleavage generates enzymatically active caspase-1, which initiates the maturation of the pro-inflammatory cytokines interleukin-1β (IL-1β) and interleukin-18 (IL-18), thereby linking intracellular stress sensing with innate-immune activation [6,7,25,26]. Beyond cytokine processing, activated caspase-1 also promotes pyroptotic cell death, a form of lytic, pro-inflammatory programmed cell death mediated by gasdermin D (GSDMD) pore formation, contributing to the amplification of inflammatory responses within metabolically dysfunctional adipose tissue [32,33,34].

The modular architecture of the NLRP3 inflammasome explains its capacity to integrate multiple metabolic stress signals into a unified inflammatory response. Rather than functioning as an isolated signalling complex, the inflammasome operates as a molecular hub in which structural organisation directly determines biological function. This property is particularly relevant in obesity, where simultaneous activation of mitochondrial stress, oxidative stress, endoplasmic reticulum stress and hypoxia converges on a common inflammasome-mediated inflammatory programme [6,7,18,25,26].

Understanding the structural organisation of the NLRP3 inflammasome provides the mechanistic basis for interpreting the sequential processes of inflammasome priming, activation and downstream inflammatory signalling discussed in the following sections.

### 7.2. DAMPs as Molecular Integrators of Metabolic Stress in Obesity

Unlike pathogen-associated molecular patterns (PAMPs), which initiate inflammatory responses during infection, the NLRP3 inflammasome in obesity is activated predominantly by endogenous damage-associated molecular patterns (DAMPs) generated within metabolically stressed adipocytes [6,7,25,26]. These molecules are not foreign to the organism; rather, they represent intracellular indicators of impaired metabolic homeostasis resulting from chronic lipid overload, mitochondrial dysfunction, oxidative stress and organelle injury. Consequently, obesity-associated inflammation should be regarded as a sterile inflammatory response driven by endogenous danger signals rather than by microbial infection.

A characteristic feature of obesity is the diversity of DAMPs capable of activating NLRP3. Saturated free fatty acids (FFAs), ceramides, extracellular ATP, mitochondrial DNA (mtDNA), cholesterol crystals, lysosomal cathepsins and reactive oxygen species (ROS) originate from distinct cellular compartments and arise through different pathogenic mechanisms [6,7,25,26,32,33]. Nevertheless, accumulating evidence indicates that these apparently unrelated signals converge on a limited number of common intracellular events that determine inflammasome activation. Thus, the biological significance of individual DAMPs lies less in their specific origin than in their ability to collectively signal a substantial loss of adipocyte homeostasis.

#### 7.2.1. Lipotoxic DAMPs: FFAs and Ceramides

Among obesity-associated danger signals, saturated FFAs and ceramides constitute the primary mediators linking chronic nutrient excess with inflammatory activation [6,7,32]. Persistent lipid overload disrupts mitochondrial oxidative metabolism, enhances ROS generation and promotes release of mitochondrial DNA into the cytoplasm. Simultaneously, oxidative stress induces dissociation of the thioredoxin-interacting protein (TXNIP) from thioredoxin, enabling direct interaction of TXNIP with the NLRP3 receptor and promoting inflammasome assembly. Rather than acting independently, these mechanisms cooperate to transform metabolic overload into a coordinated inflammatory response.

Experimental studies indicate that mitochondrial dysfunction can activate the cGAS–STING signalling pathway independently of NLRP3, providing an additional route to type I interferon and pro-inflammatory signalling [27,28]. At present, this mechanism is supported mainly by cellular and animal evidence in obesity-related settings; its magnitude, tissue specificity and clinical relevance in human adipose tissue remain to be established. Defective autophagy may likewise promote accumulation of damaged mitochondria and sustain ROS production in experimental models, suggesting a role for mitochondrial quality control in limiting inflammatory activation.

#### 7.2.2. Lysosomal and Purinergic Danger Signals

Not all DAMPs originate directly from adipocytes. Cholesterol crystals engulfed by adipose tissue macrophages destabilise lysosomes and release cathepsin B, thereby facilitating NLRP3 activation [32]. In parallel, extracellular ATP released from damaged or dying adipocytes activates the purinergic P2X7 receptor, inducing potassium efflux, a major convergent signal implicated in NLRP3 inflammasome activation across multiple stimuli [25,26,45]. Thus, lysosomal damage and purinergic signalling should be viewed as complementary components of a unified danger-sensing network rather than as independent activation pathways.

#### 7.2.3. ROS as the Convergence Point of Cellular Stress

Reactive oxygen species occupy a unique position within this network because they integrate multiple forms of metabolic stress. Mitochondrial dysfunction, ER stress, activation of nicotinamide adenine dinucleotide phosphate (NADPH) oxidase and impaired antioxidant defence all contribute to excessive ROS production [6,7,25]. Beyond causing oxidative damage, ROS function as signalling molecules that facilitate TXNIP-mediated NLRP3 activation and reinforce inflammatory gene expression through NF-κB. Consequently, oxidative stress should be regarded not simply as one of many DAMPs but as a central amplifier connecting diverse metabolic disturbances with inflammasome signalling.

#### 7.2.4. Convergence of Distinct DAMPs into a Common Inflammatory Programme

Although obesity generates numerous endogenous danger signals, current evidence indicates that they converge on a shared molecular programme, with potassium efflux representing a major trigger, characterised by potassium efflux, ASC oligomerisation, caspase-1 activation and maturation of IL-1β and IL-18 [6,7,25,26]. This convergence explains why inhibition of the NLRP3 NACHT domain suppresses inflammasome activation irrespective of the initiating DAMP. More importantly, it demonstrates that chronic inflammation in obesity is not driven by any single metabolic abnormality but by the cumulative burden of cellular stress exceeding the adaptive capacity of adipose tissue.

DAMPs should therefore be considered not as isolated inflammatory ligands but as integrated biomarkers of progressive adipocyte dysfunction. Their collective action links impaired lipid handling, mitochondrial injury, oxidative stress and immune activation into a self-reinforcing immunometabolic network that sustains chronic metaflammation throughout the course of obesity [6,7,18,25,26].

### 7.3. Caspase-1 as the Effector Hub Driving Self-Amplifying Immunometabolic Inflammation

Activation of the NLRP3 inflammasome culminates in the conversion of inactive procaspase-1 into catalytically active caspase-1, which serves as the principal effector enzyme linking intracellular danger sensing with tissue-level inflammatory responses [6,7,25,26]. Although caspase-1 is traditionally recognised for processing interleukin-1β (IL-1β) and interleukin-18 (IL-18), accumulating evidence indicates that its biological role extends far beyond cytokine maturation. In hypertrophic adipocytes, caspase-1 coordinates multiple processes that collectively transform transient metabolic stress into a persistent self-amplifying inflammatory network.

#### 7.3.1. Pyroptosis: Amplification Through DAMP Release

A major downstream consequence of caspase-1 activation in experimental systems is cleavage of GSDMD, generating the pore-forming N-terminal fragment (GSDMD-N) [32,33]. GSDMD-N oligomerisation can induce pyroptosis, a lytic inflammatory form of programmed cell death that releases ATP, HMGB1, mitochondrial DNA and IL-1α. This provides a plausible feed-forward mechanism for propagating inflammation. However, the extent to which adipocyte pyroptosis, as opposed to pyroptosis in infiltrating immune cells or other forms of adipocyte death, drives human obesity-associated adipose inflammation remains incompletely resolved.

#### 7.3.2. Metabolic Reprogramming Induced by Caspase-1

Beyond its role in pyroptosis, caspase-1 can influence adipocyte metabolism and differentiation through cytokine-dependent mechanisms and proteolytic processing of regulatory proteins [6,46]. In particular, inflammation-activated caspase-1 can cleave SIRT1 in adipose tissue, compromising mitochondrial and metabolic homeostasis and potentially increasing susceptibility to further inflammatory activation [46]. Together with IL-1β-dependent effects on adipocyte differentiation [6], these mechanisms indicate that caspase-1 not only responds to adipose tissue dysfunction but may also reinforce metabolic disturbances associated with chronic inflammation.

#### 7.3.3. Inter-Inflammasome Crosstalk

Recent evidence indicates that inflammasome pathways may interact with broader innate immune and inflammatory cell-death networks rather than functioning entirely in isolation. Studies of the NLR family CARD domain-containing protein 4 (NLRC4) inflammasome illustrate the capacity of inflammasome signalling to participate in interconnected innate immune responses [47]. The extent to which direct inter-inflammasome crosstalk contributes to persistent metaflammation in obesity remains to be clarified.

#### 7.3.4. IL-1β: From Physiological Mediator to Driver of Chronic Disease

Among the downstream mediators of inflammasome activation, IL-1β has direct translational relevance [6,7,26], sustained IL-1 signalling is implicated in β-cell dysfunction and insulin resistance, and clinical studies of IL-1 antagonism, including anakinra, have reported improvements in selected inflammatory and glycaemic or β-cell-related outcomes [34]. These intervention data support the biological relevance of the IL-1 pathway but do not, by themselves, prove that adipose-tissue NLRP3 activation is the principal upstream cause of metabolic disease in humans.

**Figure 3:**
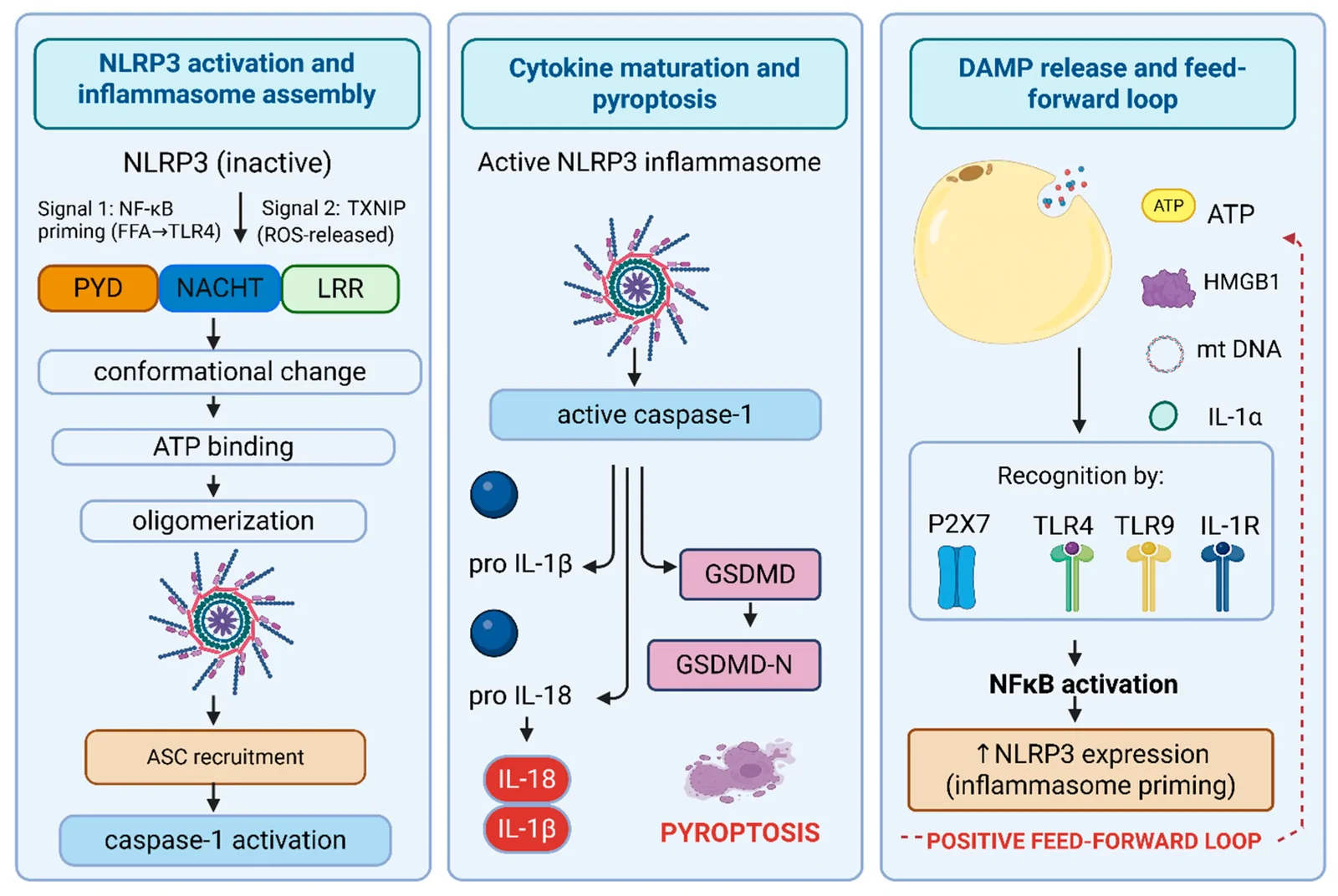
**NLRP3 inflammasome activation and caspase-1-dependent amplification in hypertrophic adipocytes**. Adipocyte hypertrophy generates converging upstream signals, including free fatty acid accumulation, mitochondrial reactive oxygen species, endoplasmic reticulum stress and DAMP release, that prime and activate the NLRP3 inflammasome. Active caspase-1 cleaves pro-IL-1β and pro-IL-18 to their mature forms, initiates gasdermin-D-dependent pyroptosis and impairs mitochondrial function, establishing a positive feed-forward inflammatory loop within dysfunctional adipose tissue. **Abbreviations:** AMPK, AMP-activated protein kinase; DAMPs, damage-associated molecular patterns; ER, endoplasmic reticulum; FFA, free fatty acid; IL-1β/18, interleukin-1β/18; mtROSs, mitochondrial reactive oxygen species; NF-κB, nuclear factor κB; NLRP3, NOD-, LRR- and pyrin domain-containing protein 3; ROS, reactive oxygen species; TXNIP, thioredoxin-interacting protein. Created in BioRender. Grudniewska, A. (2026) https://BioRender.com/pkhfv18 (accessed on 20 July 2026).

The mechanistic architecture of this self-reinforcing network is depicted in Figure 3, which illustrates caspase-1-dependent cytokine maturation, pyroptosis and the resulting positive feed-forward inflammatory loop.

Caspase-1 thus has a broader role than that of the terminal enzyme of the NLRP3 inflammasome. Through its involvement in pyroptosis, IL-1β and IL-18 maturation, impaired adipogenesis, mitochondrial dysfunction and DAMP release, caspase-1 may contribute to a self-reinforcing inflammatory loop that sustains immunometabolic dysfunction [6,7,25,26,32,33]. This integrated effector programme, illustrated in Figure 3, provides a possible link between local adipocyte stress, persistent tissue inflammation and the development of systemic metabolic disease.

## 8. Adipokines as Master Regulators of Immunometabolic Homeostasis

Adipose tissue is increasingly recognised as an active endocrine organ whose biological activity extends far beyond lipid storage. Through the secretion of adipokines, adipocytes continuously regulate systemic energy metabolism, insulin sensitivity, vascular homeostasis and innate immune responses [4,12,14]. Under physiological conditions, these mediators maintain a dynamic equilibrium between metabolic adaptation and immune surveillance, allowing adipose tissue to expand while preserving immunometabolic homeostasis.

The transition from adaptive adipocyte hyperplasia to pathological hypertrophy profoundly remodels this endocrine function. Rather than representing isolated alterations in individual adipokines, obesity induces coordinated reprogramming of the entire adipokine network [12,14,35,44]. Anti-inflammatory mediators decline, whereas adipokines promoting immune activation become increasingly dominant. This shift transforms adipose tissue from a metabolically protective endocrine organ into a chronic source of inflammatory signalling that sustains local and systemic metaflammation.

Adipokines should not be viewed simply as downstream biomarkers of adipocyte dysfunction. Current evidence indicates that they actively regulate multiple components of the inflammatory network described in previous sections, including NLRP3 inflammasome activation, macrophage recruitment, mitochondrial homeostasis, oxidative stress and insulin signalling [6,7,14,44]. Consequently, altered adipokine secretion establishes multiple positive feedback loops that reinforce adipocyte dysfunction long after the initiating metabolic stress has occurred.

Within this regulatory network, adiponectin and leptin occupy central but functionally opposing positions. Adiponectin maintains adipocyte metabolic integrity through suppression of inflammatory signalling and preservation of mitochondrial function, whereas leptin promotes innate-immune activation, macrophage polarisation and inflammasome priming [12,14,35,44]. Additional adipokines, including resistin, visfatin and chemerin, further amplify these inflammatory circuits, creating an integrated endocrine network that contributes to chronic immunometabolic dysfunction [14,48,49,50].

Adipokine remodelling is best viewed as an important component of the network linking adipocyte hypertrophy with persistent local and systemic inflammation, rather than as a stand-alone explanation for obesity-associated metabolic dysfunction [12,14,18].

### 8.1. Adiponectin—Loss of Protective Function

Adiponectin is a homotrimeric protein produced by mature adipocytes, circulating in low-molecular-weight (LMW), medium-molecular-weight (MMW) and high-molecular-weight (HMW) forms [14]. The AdipoR1/R2 receptors are coupled to the adaptor protein APPL1 [14].

Activation of AdipoR1 and AdipoR2 stimulates AMP-activated protein kinase (AMPK) through liver kinase B1 (LKB1). This signalling can inhibit mTORC1 and NF-κB activity, in part through stabilisation of IκBα, while also activating PPARα and promoting fatty acid β oxidation [12,14]. Adiponectin may further influence lipid-driven inflammation through T cadherin. Activation of this pathway stimulates ceramidase activity and promotes ceramide degradation, potentially reducing the availability of lipid signals that can activate the NLRP3 inflammasome [14].

Adiponectin may also regulate NLRP3 activation through its effects on cellular redox balance. Wang et al. demonstrated that AMPK activation following adiponectin signalling can reduce ROS production and thereby limit TXNIP release from the thioredoxin TXN complex. This provides a mechanistic link between adiponectin signalling, oxidative stress and NLRP3 inflammasome activation [51].

The adiponectin-to-leptin ratio (AdipoQ/Lep) may provide a broader measure of adipose tissue dysfunction. Both the review by Engin [29] and the study by Castela et al. [30] support its association with adipose tissue dysfunction and insulin resistance, with lower values associated with greater cardiometabolic risk. Adiponectin expression is regulated by several pathways that become disrupted during adipocyte dysfunction. NF-κB can suppress transcription of the adiponectin gene (ADIPOQ), while endoplasmic reticulum stress activates inositol-requiring enzyme 1α (IRE1α). Through non-canonical mRNA splicing, IRE1α promotes the formation of XBP1s, which has been linked to increased expression of miR 103 and miR 107 and subsequent destabilisation of ADIPOQ transcripts [14]. TNF α provides another route of suppression. Through activation of JNK and the AP 1 transcriptional pathway, TNF α can interfere with PPARγ activity, an important positive regulator of adiponectin expression in adipocytes [14,18].

These pathways may converge to create a self-sustaining inflammatory loop within dysfunctional adipose tissue. Adipocyte hypertrophy is associated with reduced adiponectin production, weakening an important endogenous brake on NF-κB signalling. Increased NF-κB activity can then promote further production of inflammatory mediators and aggravate adipocyte dysfunction. This may further suppress adiponectin through the pathways described above, reinforcing the pro-inflammatory state of adipose tissue [12,14,18].

**Figure 4:**
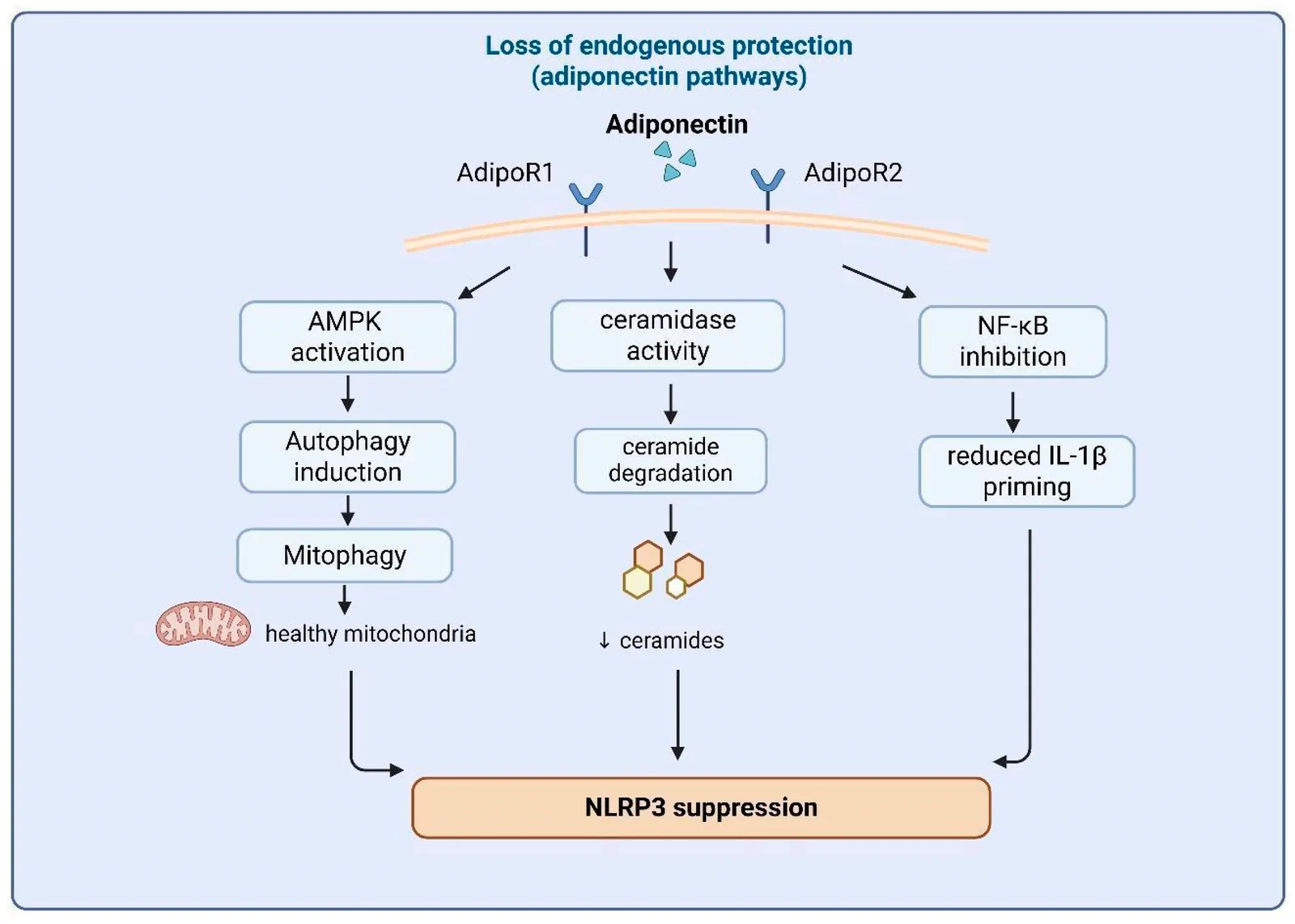
**Adiponectin-mediated suppression of NLRP3 inflammasome activity through three receptor-dependent pathways**. Adiponectin signals through AdipoR1 and AdipoR2 to engage three complementary anti-inflammatory mechanisms: (1) AdipoR1–LKB1–AMPK activation suppresses mTORC1 and stabilises IκBα, reducing NF-κB-dependent NLRP3 priming; (2) AdipoR2–PPARα activation reduces mitochondrial ROS generation, limiting TXNIP-dependent inflammasome assembly; (3) cAMP-dependent PKA activation directly inhibits NLRP3 oligomerisation. Progressive adiponectin deficiency in obesity removes all three brakes simultaneously, potentiating NLRP3-driven metaflammation. **Abbreviations:** AMPK, AMP-activated protein kinase; cAMP, cyclic adenosine monophosphate; IκBα, inhibitor of nuclear factor κB α; LKB1, liver kinase B1; mTORC1, mechanistic target of rapamycin complex 1; NF-κB, nuclear factor κB; NLRP3, NOD-, LRR- and pyrin domain-containing protein 3; PKA, protein kinase A; PPARα, peroxisome proliferator-activated receptor α; ROS, reactive oxygen species; TXNIP, thioredoxin-interacting protein. Created in BioRender. Grudniewska, A. (2026). BioRender. https://BioRender.com/kgqaeji (accessed on 21 July 2026).

The three parallel suppressive pathways are summarised in Figure 4. Adiponectin signals through AdipoR1 and AdipoR2 receptors via: (1) AdipoR1-mediated AMPK activation inducing autophagy and selective mitophagy of damaged mitochondria, thereby reducing ROS generation and TXNIP-dependent NLRP3 activation; (2) AdipoR2-mediated ceramidase activity promoting ceramide degradation, removing a key NLRP3-activating lipid ligand; and (3) inhibition of NF-κB signalling reducing transcriptional priming of NLRP3 and pro-IL-1β. In obesity, impaired adiponectin signalling may weaken these complementary protective mechanisms, thereby favouring NLRP3-associated inflammatory activation.

### 8.2. Leptin—Molecular Mechanisms of Pro-Inflammatory Action

Leptin exerts its biological effects through the long isoform of the leptin receptor (OB-Rb), coupled to the tyrosine kinase Janus kinase 2 (JAK2). Upon JAK2 activation, the transcription factors signal transducer and activator of transcription 3 (STAT3) and signal transducer and activator of transcription 5 (STAT5) and the phosphatase Src homology region 2-containing phosphatase 2 (SHP-2) are phosphorylated and activated, initiating a signalling cascade regulating both neuroendocrine and immunological functions [12,14].

In immune cells, including macrophages infiltrating adipose tissue, leptin can activate the phosphoinositide 3-kinase (PI3K) → Akt → mTORC1 pathway and promote a shift towards a pro-inflammatory M1 phenotype. Leptin-activated M1 macrophages produce higher amounts of TNF α, IL- 6, interleukin 12 (IL- 12) and IL-1β [14,52]. Leptin can also increase TLR4 expression on macrophages, potentially enhancing their responsiveness to free fatty acids and lipopolysaccharides, which are important inflammatory stimuli in obesity. Through JAK2 and STAT3 signalling, leptin may additionally increase the expression of NLRP3 and pro IL-1β, thereby favouring inflammasome priming and potentially facilitating subsequent activation [6,7,14].

A clinically relevant feature of obesity is the coexistence of high circulating leptin concentrations with reduced responsiveness to some of its effects. This phenomenon, commonly referred to as leptin resistance, is particularly evident in the hypothalamus, where chronic leptin exposure can induce suppressor of cytokine signalling 3 (SOCS3), an endogenous inhibitor of JAK STAT signalling. As a result, the anorexigenic effects of leptin become attenuated despite persistently elevated hormone concentrations. This central resistance does not necessarily prevent leptin signalling in peripheral tissues. Pro-inflammatory responses in macrophages, adipocytes and endothelial cells may therefore persist even when hypothalamic leptin sensitivity is reduced [14,53].

**Figure 5:**
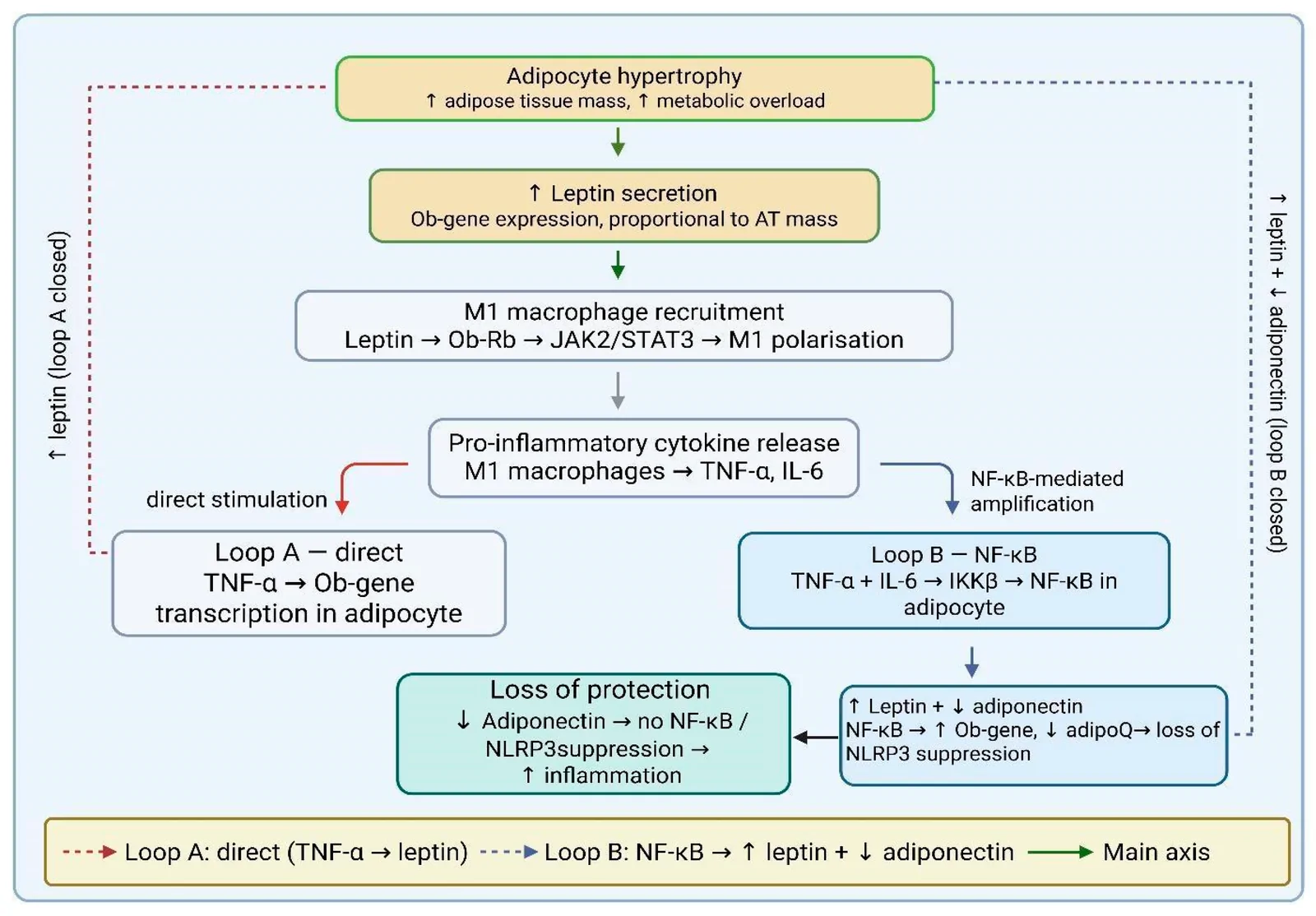
**Self-amplifying inflammatory loop linking leptin signalling with macrophage activation in hypertrophic adipose tissue**. Increased leptin signalling through OB-Rb activates JAK2/STAT3 and promotes a pro-inflammatory macrophage phenotype, with increased TNF-α and IL-6 production. These mediators activate NF-κB and NLRP3 signalling, further enhancing leptin production and sustaining local inflammation. This reciprocal interaction contributes to the persistence of adipose tissue inflammation in obesity. **Abbreviations:** IKKβ, inhibitor of nuclear factor κB kinase β; IL-6, interleukin-6; JAK2, Janus kinase 2; M1/M2, macrophage polarisation phenotypes; NF-κB, nuclear factor κB; NLRP3, NOD-, LRR- and pyrin domain-containing protein 3; OB-Rb, leptin receptor long isoform; STAT3, signal transducer and activator of transcription 3; TNF-α, tumour necrosis factor α. Created in BioRender. Grudniewska, A. (2026). BioRender. https://BioRender.com/83o0ly9 (accessed on 3 July 2026).

The proposed self-amplifying loop is illustrated in Figure 5. Adipocyte hypertrophy is associated with increased leptin production, which can promote monocyte recruitment and M1 macrophage polarisation through the long form of the leptin receptor (OB-Rb) and JAK2 STAT3 signalling. Activated macrophages release inflammatory mediators such as TNF α and IL- 6, which may further increase leptin production through complementary pathways. In one pathway, TNF α can directly stimulate leptin gene expression in adipocytes. In another, TNF α and IL- 6 activate IKKβ and NF-κB signalling, which may further promote leptin expression while suppressing adiponectin. Reduced adiponectin signalling then weakens an important endogenous restraint on NLRP3 and NF-κB activity, providing an additional mechanism through which adipose tissue inflammation may become self-reinforcing.

### 8.3. Resistin, Visfatin and Chemerin

Among the remaining adipokines with documented pro-inflammatory activity, resistin, visfatin and chemerin warrant attention; their mechanisms complement and reinforce the inflammatory pathways described above.

Resistin, also known as found in inflammatory zone 3 (FIZZ3), signals through Toll-like receptor 4 (TLR4) and cyclase-associated protein 1 (CAP 1). TLR4 activation engages the MyD88, IRAK4 and TRAF6 signalling cascade and can also activate p38 mitogen-activated protein kinase (p38 MAPK), promoting the expression and stability of pro-inflammatory mediators [14,48]. NF-κB activation increases the production of TNF α, IL- 6 and IL- 12 and may therefore contribute to the priming of the NLRP3 inflammasome and maintenance of the inflammatory environment in obese adipose tissue. CAP 1-dependent signalling can additionally activate the cAMP and PKA pathway, increasing MCP 1 production and potentially promoting monocyte recruitment and endothelial adhesion. These effects may extend the influence of resistin beyond adipose tissue to the vascular compartment [48].

Visfatin, also known as nicotinamide phosphoribosyltransferase (NAMPT), illustrates how the biological effects of an adipose tissue factor can depend on its cellular location. Intracellular NAMPT (iNAMPT) is a key enzyme in the NAD^+^ salvage pathway and contributes to maintenance of NAD^+^/SIRT1-dependent metabolic signalling [54]. In contrast, extracellular NAMPT (eNAMPT), which can be released by hypertrophic adipocytes and infiltrating macrophages, has been associated with pro-inflammatory signalling through TLR4 and NF-κB and with increased production of IL-1β and IL-18 [49]. This compartment-dependent behaviour makes NAMPT particularly interesting in the context of obesity. As adipocyte hypertrophy alters both intracellular metabolism and extracellular signalling, the balance between these two forms may shift towards a more inflammatory environment [49,54].

Chemerin is a chemotactic protein produced predominantly by adipose tissue and signals mainly through the G protein-coupled receptor CMKLR1. Its effects may involve both intracellular inflammatory signalling and recruitment of immune cells. CMKLR1 activation can promote IκB phosphorylation and degradation, facilitating NF-κB activation and increasing the expression of inflammatory mediators such as TNF α, IL-6 and IL-1β [14,50]. Because these cytokines can contribute to NLRP3 priming and macrophage activation, chemerin may thereby reinforce local adipose tissue inflammation.

Chemerin also participates in the recruitment of immune cells, including plasmacytoid dendritic cells, myeloid dendritic cells, macrophages and natural killer cells. Recruitment of plasmacytoid dendritic cells to visceral adipose tissue may increase local type I interferon signalling and further influence inflammatory gene expression [50]. Chemerin activity is regulated by proteolytic processing involving components of the coagulation and complement systems. This provides a potential link between adipose tissue inflammation and the prothrombotic environment associated with obesity, in which altered coagulation may favour the generation of more active chemerin forms and thereby contribute to persistence of inflammatory signalling [14,50].

Current evidence supports investigating adipose tissue functional characteristics alongside adipose tissue quantity. Translation into routine clinical practice will require prospective validation showing that candidate measures of adipose tissue dysfunction improve risk prediction or treatment selection beyond established anthropometric and metabolic parameters.

## 9. Discussion

The main contribution of this review is an integrative, evidence-weighted model that links adipose tissue remodelling with cellular stress, endocrine change and immune activation without treating any single molecular pathway as sufficient. The proposed ‘expandability threshold’ is therefore a conceptual organising device rather than a novel fixed biological cut-off, and the detailed treatment of NLRP3 is intended to illustrate one well-developed inflammatory node within a broader network.

Hyperplasia, hypertrophy, fibrosis, hypoxia and inflammation may coexist, and their relative contribution varies by adipose depot, sex, age, genetic background and stage of obesity. The framework should therefore be interpreted as a set of interacting processes with possible feed-forward relationships rather than as an obligatory linear chronology [4,5,6,7,13,14,16,18,19].

Within this network, JNK, IKKβ/NF-κB and TLR4 signalling connect nutrient and inflammatory stress with altered insulin action, although ER stress, mitochondrial stress and oxidative stress provide overlapping upstream inputs. NLRP3–caspase-1 signalling can integrate several danger signals and amplify IL-1-family responses, whereas cGAS–STING represents an additional, less clinically validated route linking mitochondrial damage to innate immunity [6,7,8,14,18,19,24,25,26,27,28]. This positioning avoids assigning NLRP3 a uniquely dominant role that is not established in human obesity.

The evidence base also differs across these mechanisms. Human data are strongest for adipocyte hypertrophy, visceral adiposity, altered adipokines, macrophage accumulation, inflammation and insulin resistance, whereas detailed intracellular sequences involving TXNIP, gasdermin-D-dependent pyroptosis and cGAS–STING remain more dependent on experimental models. This distinction is reflected in Table 1 and should guide interpretation of therapeutic targets.

The term ‘adipose tissue quality’ is used here as a biological umbrella encompassing cellular morphology and expandability, adipogenesis, vascular and extracellular matrix integrity, inflammatory state, adipokine secretion and metabolic/lipid-buffering function. It should not be equated with any single circulating biomarker or imaging measure. Current clinical and research tools capture only selected dimensions of this construct.

The proposed framework has potential translational implications, but its clinical use is currently limited. BMI, waist circumference and standard cardiometabolic variables remain established tools for routine assessment. Imaging of visceral fat may refine phenotyping in selected settings, whereas the adiponectin-to-leptin ratio, perivascular adipose imaging and molecular inflammatory signatures remain candidate or research measures whose incremental predictive value requires prospective validation [29,30,31]. Table 1 therefore separates established clinical parameters from research biomarkers and experimental readouts.

The therapeutic implications should likewise be interpreted cautiously. Weight reduction remains a central component of obesity management, but the mechanisms reviewed here suggest that improvement in adipose tissue function may represent an additional component of the metabolic benefit associated with effective treatment. Strategies that reduce adipocyte hypertrophy, improve adipogenic capacity, modulate adipokine signalling or attenuate excessive inflammasome activation may therefore influence complementary aspects of adipose tissue dysfunction [6,13,18]. Pharmacological approaches, including PPARγ agonism and incretin-based therapies, provide relevant examples, although the extent to which their clinical effects are mediated specifically through restoration of adipose tissue function remains to be determined.

Adiposity and adipose tissue dysfunction should not be considered competing therapeutic concepts. Reduction in excess adiposity can decrease upstream drivers of cellular stress, whereas interventions directed at inflammatory, metabolic or endocrine pathways may modify mechanisms that sustain dysfunction. The relative contribution of these effects varies by intervention and remains an active area of investigation. This distinction provides the conceptual basis for subsequent parts of the Prometheus’s Fire series, which address inter-organ inflammatory propagation and therapeutic modulation in greater detail.

## 10. Limitations

Several limitations of the present review should be acknowledged. First, this is a narrative, state-of-the-art synthesis rather than a systematic review; study selection was guided by mechanistic relevance, methodological quality and translational significance, and formal risk-of-bias scoring was not performed. Although the search strategy and selection principles are reported to improve reproducibility, some selection bias remains possible. Second, the proposed model integrates evidence obtained across cell culture studies, animal models, human observational studies and clinical trials. This breadth allows construction of an integrative framework, but causal effects demonstrated experimentally cannot automatically be assumed to have the same magnitude or sequence in humans.

Third, many mechanisms discussed in relation to adipose tissue expandability and inflammasome activation have been investigated predominantly in specific adipose tissue depots or experimental models. Adipose tissue is highly heterogeneous, and biological processes in visceral, subcutaneous, perivascular and other depots may differ substantially. Consequently, the proposed sequence from impaired expandability to hypertrophy, cellular stress and chronic inflammation should be regarded as a conceptual framework rather than a universally identical progression across all individuals and adipose tissue compartments.

Fourth, the concept of an adipose tissue expandability threshold remains difficult to operationalise clinically. There is currently no validated method for directly measuring individual expandability or identifying a precise transition between adaptive and pathological remodelling. Candidate biomarkers, including adipokine ratios, circulating lipid species and molecular inflammatory markers, require standardisation and prospective validation before routine use.

Finally, the emphasis placed on the NLRP3 inflammasome should not imply that it is the sole driver of obesity-associated inflammation. Other stress-sensing and inflammatory pathways, including cGAS–STING signalling, may interact with or operate alongside inflammasome pathways. In addition, cardiometabolic risk is multifactorial and reflects fat distribution, ectopic lipid deposition, genetics, age, sex, lifestyle and established metabolic risk factors as well as adipose tissue biology.

These limitations reflect the complexity and heterogeneity of obesity-associated immunometabolic disease and highlight the need for longitudinal human studies capable of examining several interconnected mechanisms simultaneously.

## 11. Future Perspectives

Several important questions arising from the present framework remain unresolved. A major priority is the identification of reliable biomarkers capable of detecting early deterioration in adipose tissue function before the development of overt metabolic complications. Prospective studies should therefore evaluate whether markers of adipokine balance, lipid toxicity, inflammasome activity and mitochondrial stress can identify individuals approaching the transition from adaptive remodelling towards persistent adipose tissue dysfunction.

Further research is also needed to define the biological determinants of individual adipose tissue expandability. These are likely to reflect complex interactions among developmental factors, sex, ageing, adipose tissue depot characteristics and environmental influences. Whether different adipose tissue depots possess distinct functional thresholds and how these differences contribute to individual obesity phenotypes remain important questions.

Longitudinal human studies incorporating detailed phenotyping of adipose tissue depots, together with emerging approaches such as spatial transcriptomics, single-cell RNA sequencing, proteomics, metabolomics and advanced imaging, may help clarify these mechanisms. Such approaches could also establish whether changes in molecular markers precede detectable alterations in conventional clinical measures and thereby identify earlier stages at which intervention may be most effective.

An additional area for future investigation concerns the interaction between the NLRP3 inflammasome and other cellular stress-sensing pathways. Emerging evidence suggests that mitochondrial and cellular damage may activate inflammatory mechanisms extending beyond the inflammasome itself, including cGAS–STING signalling [28]. Understanding how these pathways interact within different adipose tissue depots and during different stages of obesity may provide a more complete picture of the processes determining whether local metabolic stress remains adaptive or progresses towards chronic inflammation.

The framework developed here also raises important therapeutic questions that are addressed further in Parts II and III of the Prometheus’s Fire series. In particular, it remains to be determined to what extent successful obesity treatment should be understood solely in terms of reducing adiposity and to what extent it should also aim to restore adipose tissue immunometabolic function. Future studies will need to establish whether interventions acting through complementary mechanisms can provide additional benefits by simultaneously reducing the upstream drivers of dysfunction and attenuating the self-reinforcing inflammatory processes that contribute to its persistence.

## 12. Conclusions

Current evidence supports an integrative model in which adipose tissue mass and distribution interact with depot-specific adaptive capacity and biological function. Hyperplasia and hypertrophy can coexist, and the balance between them varies with sex, age, genetics, adipose depot and obesity stage. When storage demand exceeds local adipogenic, vascular and structural adaptation, hypertrophic and stress-related processes may become more prominent and contribute to immunometabolic dysfunction.

The novelty of this review lies primarily in organising these established mechanisms into an evidence-weighted network rather than proposing a new isolated pathway. NLRP3 is positioned within a broader system that includes JNK, IKKβ/NF-κB, TLR4, ER and mitochondrial stress, oxidative stress and emerging cGAS–STING signalling. Human evidence is not equally strong for every component, and detailed inflammasome, pyroptotic and cGAS–STING mechanisms remain more dependent on experimental models.

Clinically, ‘adipose tissue quality’ remains a multidimensional biological construct rather than a validated diagnostic variable. Established anthropometric and metabolic measures should remain the basis of assessment. Visceral imaging, adiponectin-to-leptin ratios, circulating ceramides, perivascular or fibrotic adipose imaging and molecular inflammatory readouts differ substantially in validation and availability and should not be treated as interchangeable markers of tissue function.

The framework developed in this review provides the mechanistic foundation for the subsequent parts of the Prometheus’s Fire series. Part II, ‘The Hydra of Inflammation’, extends the concept of local adipose tissue dysfunction to self-amplifying inter-organ inflammatory processes, whereas Part III, ‘The Forge of Hephaestus’, explains how lifestyle-based and pharmacological interventions may modify these interconnected mechanisms and contribute to the restoration of immunometabolic homeostasis.

The transition from adaptive adipose tissue expansion towards dysfunctional hypertrophy is an important contributing process in obesity-associated immunometabolic disease, but it operates within a broader multifactorial risk architecture. Clarifying which elements of this framework are causal and clinically measurable in humans will be essential for translating adipose tissue biology into more biologically informed risk stratification and treatment.

## Figures and Tables

**Table 1:** Evidence hierarchy, candidate indicators and current clinical relevance of key mechanisms in adipose tissue dysfunction.

Mechanism/ Domain	Strength and Source of Evidence	Candidate Indicators	Current Clinical Relevance	Status/Representative References
Adipose tissue amount and distribution	Human observational and clinical evidence; established association with cardiometabolic outcomes	BMI; waist circumference; visceral adipose tissue by CT/MRI when clinically indicated	BMI and waist circumference are established clinical measures; imaging is used selectively. These measures do not directly quantify tissue functional capacity.	Established clinical parameters [11,13,53]
Adipocyte hypertrophy/limited adaptive expansion	Human adipose-tissue studies plus strong experimental support; direct expandability threshold not validated clinically	Adipocyte size from biopsy; adipogenic capacity; depot-specific morphology	Biopsy-based measures are research tools; no validated routine test of adipose tissue expandability exists.	Translational/ research [5,13,48,49]
Adipokine dysregulation	Human observational evidence with mechanistic experimental support	Adiponectin; leptin; adiponectin-to-leptin ratio	Adiponectin and leptin are measurable, but the ratio lacks standardised clinical thresholds and proven incremental utility for routine risk stratification.	Candidate biomarker [29,30]
Visceral, perivascular and fibrotic adipose phenotypes	Human imaging evidence is strongest for visceral adiposity; perivascular and fibrosis-oriented phenotypes have more limited/specialist validation	Visceral fat volume by CT/MRI; perivascular imaging phenotypes; research imaging of fibrosis/remodelling	Visceral imaging can refine phenotyping when clinically indicated; perivascular and fibrosis-focused measures remain mainly specialist/research tools.	Mixed: established association to emerging imaging [31]
Mitochondrial/ER stress, oxidative stress and hypoxia	Convergent experimental evidence with supportive human tissue observations; causal ordering in humans uncertain	ROS/redox signatures; mitochondrial function; stress-response proteins; HIF-1α-related signatures	No standardised circulating or tissue panel is validated for routine clinical risk stratification.	Research biomarkers [6,7,16,17,18,19]
NLRP3–caspase-1–IL-1 axis	Strong causal evidence in cellular/animal models; human tissue associations and indirect clinical support from IL-1 pathway intervention	NLRP3/caspase-1 activity; IL-1β/IL-18; inflammasome-related signatures	Biologically and therapeutically relevant, but no validated NLRP3-based test is recommended for routine obesity risk stratification.	Translational; not routine [17,18,20,21,36]
Pyroptosis/gasdermin D	Predominantly experimental; contribution of adipocyte versus immune-cell pyroptosis in humans remains uncertain	GSDMD cleavage; pyroptosis-associated DAMPs	Experimental readouts; not validated clinical biomarkers.	Experimental/ emerging [32,33]
cGAS–STING signalling	Predominantly cellular and animal evidence in obesity/metabolic models; limited human validation	cGAS/STING activation; type I interferon signatures; cytosolic mtDNA-related measures	Mechanistically plausible research pathway; no established clinical biomarker or obesity-specific indication.	Experimental/ emerging [27,28]
Circulating ceramides/lipidomic signatures	Growing human observational and prognostic evidence in cardiometabolic disease; adipose-tissue specificity is limited	Plasma ceramide species; lipidomic risk scores	Potential prognostic biomarkers in selected research/clinical contexts, but not validated as a specific measure of adipose tissue quality; availability and standardisation remain limited.	Emerging biomarker; not adipose-specific

## Data Availability

No new data were created or analysed in this study. Data sharing is not applicable to this article.

## Abbreviations

The following abbreviations are used in this manuscript: ADIPOQ, adiponectin gene; AdipoQ/Lep, adiponectin-to-leptin ratio; AdipoR1/2, adiponectin receptor 1/2; AMPK, AMP-activated protein kinase; AP-1, activator protein 1; APPL1, adaptor protein, phosphotyrosine interacting with PH domain and leucine zipper 1; ASC, apoptosis-associated speck-like protein containing a caspase recruitment domain; ATP, adenosine triphosphate; BMI, body mass index; CAP 1, cyclase-associated protein 1; CARD, caspase activation and recruitment domain; cGAS–STING, cyclic GMP–AMP synthase–stimulator of interferon genes; CLS, crown-like structures; CMKLR1, chemokine-like receptor 1; DAMP, damage-associated molecular pattern; eNAMPT, extracellular nicotinamide phosphoribosyltransferase; ER, endoplasmic reticulum; FFA, free fatty acid; FIZZ3, found in inflammatory zone 3; GSDMD, gasdermin D; GSDMD-N, gasdermin D N-terminal fragment; HIF-1α, hypoxia-inducible factor 1α; HMGB1, high-mobility group box 1; HMW, high-molecular-weight; hs-CRP, high-sensitivity C-reactive protein; IKKβ, inhibitor of nuclear factor κB kinase β; IL, interleukin; iNAMPT, intracellular nicotinamide phosphoribosyltransferase; IP3R, inositol 1,4,5-trisphosphate receptor; IRAK4, interleukin-1 receptor-associated kinase 4; IRE1α, inositol-requiring enzyme 1α; IRS-1, insulin receptor substrate 1; IκBα, inhibitor of nuclear factor κB α; JAK2, Janus kinase 2; JNK, c-Jun N-terminal kinase; LKB1, liver kinase B1; LMW, low-molecular-weight; LRR, leucine-rich repeat; M1/M2, macrophage polarisation phenotypes; MAPK, mitogen-activated protein kinase; MASLD, metabolic dysfunction-associated steatotic liver disease; MCP-1, monocyte chemoattractant protein 1; MHO, metabolically healthy obesity; miR, microRNA; MMW, medium-molecular-weight; mtDNA, mitochondrial DNA; mTORC1, mechanistic target of rapamycin complex 1; MUO, metabolically unhealthy obesity; MyD88, myeloid differentiation primary response 88; NACHT, nucleotide-binding and oligomerization domain; NAD, nicotinamide adenine dinucleotide; NADPH, nicotinamide adenine dinucleotide phosphate; NAMPT, nicotinamide phosphoribosyltransferase; NF-κB, nuclear factor κB; NLRC4, NLR family CARD domain-containing protein 4; NLRP3, NOD-, LRR- and pyrin domain-containing protein 3; OB-Rb, long isoform of the leptin receptor; P2X7, purinergic receptor P2X7; PAMP, pathogen-associated molecular pattern; PI3K, phosphoinositide 3-kinase; PKA, protein kinase A; PPARα, peroxisome proliferator-activated receptor α; PPARγ, peroxisome proliferator-activated receptor γ; PYD, pyrin domain; ROSs, reactive oxygen species; SHP-2, Src homology region 2-containing phosphatase 2; SIRT1, sirtuin 1; SOCS3, suppressor of cytokine signalling 3; STAT3, signal transducer and activator of transcription 3; STAT5, signal transducer and activator of transcription 5; T2DM, type 2 diabetes mellitus; TLR, Toll-like receptor; TNF-α, tumour necrosis factor α; TRAF6, tumour necrosis factor receptor-associated factor 6; TXN, thioredoxin; TXNIP, thioredoxin-interacting protein; VEGF, vascular endothelial growth factor; XBP1s, spliced X-box-binding protein 1.

## References

[1] World Health Organization (2025). Obesity and Overweight. https://www.who.int/news-room/fact-sheets/detail/obesity-and-overweight.

[2] Cypess A.M. (2022). Reassessing Human Adipose Tissue. N. Engl. J. Med..

[3] Xu S., Lu F., Gao J., Yuan Y. (2024). Inflammation-mediated metabolic regulation in adipose tissue. Obes. Rev..

[4] Lumeng C.N., Bodzin J.L., Saltiel A.R. (2007). Obesity induces a phenotypic switch in adipose tissue macrophage polarization. J. Clin. Investig..

[5] Weisberg S.P., McCann D., Desai M., Rosenbaum M., Leibel R.L., Ferrante A.W. (2003). Obesity is associated with macrophage accumulation in adipose tissue. J. Clin. Investig..

[6] Stienstra R., Joosten L.A.B., Koenen T., van Tits B., van Diepen J.A., van den Berg S.A., Rensen P.C.N., Voshol P.J., Fantuzzi G., Hijmans A. (2010). The inflammasome-mediated caspase-1 activation controls adipocyte differentiation and insulin sensitivity. Cell Metab..

[7] Vandanmagsar B., Youm Y.H., Ravussin A., Galgani J.E., Stadler K., Mynatt R.L., Ravussin E., Stephens J.M., Dixit V.D. (2011). The NLRP3 inflammasome instigates obesity-induced inflammation and insulin resistance. Nat. Med..

[8] Scherer P.E. (2006). Adipose tissue: From lipid storage compartment to endocrine organ. Diabetes.

[9] Ahmadian M., Suh J.M., Hah N., Liddle C., Atkins A.R., Downes M., Evans R.M. (2013). PPARγ signaling and metabolism: The good, the bad and the future. Nat. Med..

[10] Rosen E.D., MacDougald O.A. (2006). Adipocyte differentiation from the inside out. Nat. Rev. Mol. Cell Biol..

[11] Bray G.A., Kim K.K., Wilding J.P.H., World Obesity Federation (2017). Obesity: A chronic relapsing progressive disease process. A position statement of the World Obesity Federation. Obes. Rev..

[12] Taylor E.B. (2021). The complex role of adipokines in obesity, inflammation, and autoimmunity. Clin. Sci..

[13] Longo M., Zatterale F., Naderi J., Parrillo L., Formisano P., Raciti G.A., Beguinot F., Miele C. (2019). Adipose tissue dysfunction as determinant of obesity-associated metabolic complications. Int. J. Mol. Sci..

[14] Ouchi N., Parker J.L., Lugus J.J., Walsh K. (2011). Adipokines in inflammation and metabolic disease. Nat. Rev. Immunol..

[15] Blüher M. (2020). Metabolically healthy obesity. Endocr. Rev..

[16] Ye J. (2011). Adipose tissue vascularization: Its role in chronic inflammation. Curr. Diab. Rep..

[17] Halberg N., Wernstedt-Asterholm I., Scherer P.E. (2008). The adipocyte as an endocrine cell. Endocrinol. Metab. Clin. N. Am..

[18] Hotamisligil G.S. (2006). Inflammation and metabolic disorders. Nature.

[19] Sun K., Halberg N., Khan M., Magalang U.J., Scherer P.E. (2013). Selective inhibition of hypoxia-inducible factor 1α ameliorates adipose tissue dysfunction. Mol. Cell. Biol..

[20] White U. (2023). Adipose tissue expansion in obesity, health, and disease. Front. Cell Dev. Biol..

[21] Vishvanath L., Gupta R.K. (2019). Contribution of adipogenesis to healthy adipose tissue expansion in obesity. J. Clin. Investig..

[22] Sun K., Kusminski C.M., Scherer P.E. (2011). Adipose tissue remodeling and obesity. J. Clin. Investig..

[23] Corvera S., Solivan-Rivera J., Loureiro Z.Y. (2022). Angiogenesis in adipose tissue and obesity. Angiogenesis.

[24] Samuel V.T., Shulman G.I. (2012). Mechanisms for insulin resistance: Common threads and missing links. Cell.

[25] He Y., Hara H., Núñez G. (2016). Mechanism and regulation of NLRP3 inflammasome activation. Trends Biochem. Sci..

[26] Dixit V.D. (2013). NLRP3 inflammasome activation in type 2 diabetes: Is it clinically relevant?. Diabetes.

[27] Zhao L., Tuerxunaili L., Shi G., Wu M., Jiao Y. (2026). Adipose tissue-specific Nrf2 knockdown inhibits the cGAS-STING pathway to attenuate inflammation in obese mice. Front. Endocrinol..

[28] He W., Mu X., Wu X., Liu Y., Deng J., Liu Y., Han F., Nie X. (2024). cGAS-STING pathway: A therapeutic target in diabetes and its complications. Burns Trauma.

[29] Engin A. (2024). Adiponectin resistance in obesity: Adiponectin leptin/insulin interaction. Adv. Exp. Med. Biol..

[30] Castela I., Morais J., Barreiros-Mota I., Silvestre M.P., Marques C., Rodrigues C., Ismael S., Araújo J.R., Ângelo-Dias M., Martins C. (2023). Decreased adiponectin/leptin ratio relates to insulin resistance in adults with obesity. Am. J. Physiol. Endocrinol. Metab..

[31] Lin A., Dey D., Wong D.T.L., Nerlekar N. (2019). Perivascular adipose tissue and coronary atherosclerosis: From biology to imaging phenotyping. Curr. Atheroscler. Rep..

[32] Wani K., AlHarthi H., Alghamdi A., Sabico S., Al-Daghri N.M. (2021). Role of NLRP3 inflammasome activation in obesity-mediated metabolic disorders. Int. J. Environ. Res. Public Health.

[33] Li X., Zhang Z., Han Y., Zhang M. (2025). NLRP3 inflammasome and pyroptosis: Implications in inflammation and multisystem disorders. PeerJ.

[34] Meier D.T., de Paula Souza J., Donath M.Y. (2025). Targeting the NLRP3 inflammasome–IL-1β pathway in type 2 diabetes and obesity. Diabetologia.

[35] Zhu L., Liu L. (2022). New insights into the interplay among autophagy, the NLRP3 inflammasome and inflammation in adipose tissue. Front. Endocrinol..

[36] Spalding K.L., Arner E., Westermark P.O., Bernard S., Buchholz B.A., Bergmann O., Blomqvist L., Hoffstedt J., Näslund E., Britton T. (2008). Dynamics of fat cell turnover in humans. Nature.

[37] Von Bank H., Kirsh C., Simcox J. (2021). Aging adipose: Depot location dictates age-associated expansion and dysfunction. Ageing Res. Rev..

[38] Virtue S., Vidal-Puig A. (2010). Adipose tissue expandability, lipotoxicity and the metabolic syndrome—An allostatic perspective. Biochim. Biophys. Acta.

[39] Trayhurn P. (2013). Hypoxia and adipose tissue function and dysfunction in obesity. Physiol. Rev..

[40] Fu Y., Luo N., Klein R.L., Garvey W.T. (2005). Adiponectin promotes adipocyte differentiation, insulin sensitivity, and lipid accumulation. J. Lipid Res..

[41] Blüher M. (2009). Adipose tissue dysfunction in obesity. Exp. Clin. Endocrinol. Diabetes.

[42] Stanford K.I., Middelbeek R.J.W., Goodyear L.J. (2015). Exercise effects on white adipose tissue: Beiging and metabolic adaptations. Diabetes.

[43] Min S.Y., Learnard H., Kant S., Gealikman O., Rojas-Rodriguez R., DeSouza T., Desai A., Keaney J.F., Corvera S., Craige S.M. (2019). Exercise rescues gene pathways involved in vascular expansion and promotes functional angiogenesis in subcutaneous white adipose tissue. Int. J. Mol. Sci..

[44] Ma E.B., Javaid H.M.A., Jung D.H., Park J.H., Huh J.Y. (2022). Gasdermin D deficiency does not protect mice from high-fat diet-induced glucose intolerance and adipose tissue inflammation. Mediat. Inflamm..

[45] Horng T. (2014). Calcium signaling and mitochondrial destabilization in the triggering of the NLRP3 inflammasome. Trends Immunol..

[46] Chalkiadaki A., Guarente L. (2012). High-fat diet triggers inflammation-induced cleavage of SIRT1 in adipose tissue to promote metabolic dysfunction. Cell Metab..

[47] Sundaram B., Kanneganti T.D. (2021). Advances in Understanding Activation and Function of the NLRC4 Inflammasome. Int. J. Mol. Sci..

[48] Benomar Y., Taouis M. (2019). Molecular mechanisms underlying obesity-induced hypothalamic inflammation and insulin resistance: Pivotal role of resistin/TLR4 pathways. Front. Endocrinol..

[49] Romacho T., Valencia I., Ramos-González M., Vallejo S., López-Esteban M., Lorenzo O., Cannata P., Romero A., San Hipólito-Luengo A., Gómez-Cerezo J.F. (2020). Visfatin/eNampt induces endothelial dysfunction in vivo: A role for Toll-Like Receptor 4 and NLRP3 inflammasome. Sci. Rep..

[50] Ernst M.C., Sinal C.J. (2010). Chemerin: At the crossroads of inflammation and obesity. Trends Endocrinol. Metab..

[51] Wang F., Liu Y., Yang W., Yuan J., Mo Z. (2018). Adiponectin inhibits NLRP3 inflammasome by modulating the AMPK-ROS pathway. Int. J. Clin. Exp. Pathol..

[52] Pereira S., Cline D.L., Glavas M.M., Covey S.D., Kieffer T.J. (2021). Tissue-specific effects of leptin on glucose and lipid metabolism. Endocr. Rev..

[53] Engin A. (2024). The mechanism of leptin resistance in obesity and therapeutic perspective. Adv. Exp. Med. Biol..

[54] Yamaguchi S., Yoshino J. (2017). Adipose tissue NAD^+^ biology in obesity and insulin resistance: From mechanism to therapy. BioEssays.

